# Body perceptions, occupations, eating attitudes, and behaviors emerged during the pandemic: An exploratory cluster analysis of eaters profiles

**DOI:** 10.3389/fpsyg.2022.949373

**Published:** 2022-12-05

**Authors:** Johana Monthuy-Blanc, Giulia Corno, Stéphane Bouchard, Marie-Josée St-Pierre, Francisca Bourbeau, Leïla Mostefa-Kara, Émie Therrien, Michel Rousseau

**Affiliations:** ^1^Groupe de Recherche Loricorps, Université du Québec à Trois-Rivières, Trois-Rivières, QC, Canada; ^2^Research Center of Mental Health University Institute of Montreal, Montreal (CR-IUSMM), Montréal, QC, Canada; ^3^Chaire de Recherche du Canada en Cyberpsychologie Clinique, Université du Québec en Outaouais, Gatineau, QC, Canada

**Keywords:** intuitive eating, dysfunctional attitudes and eating behaviors, COVID–19, incongruent-perceptual eater

## Abstract

**Introduction:**

COVID-19 pandemic negatively impacted people’s mental and physical health. Three areas have been significantly impacted, among others: eating-related behaviors, occupational balance, and exposure to self-image due to videoconferencing. This study aims to explore and document eaters profiles that were reported during the pandemic in the general Canadian population using a holistic perspective, including body perceptions, attitudes, and eating behaviors (i.e., body image, behaviors, attitudes, and motivations regarding food), and occupations (i.e., physical activity and cooking).

**Methods:**

This cross-sectional study was conducted from May to September 2020. Two hundred and seventy-three Canada’s residents, French speaking of 18 years of age and older, participated in an online survey on behaviors, attitudes, and motivations regarding food and eating as well as body image and occupations during the COVID-19 pandemic. A hierarchical cluster analysis was used to determine the eaters profiles. One-way ANOVA and Chi-square test were conducted to differentiate occupational characteristics between eaters profiles.

**Results:**

Three distinctive profiles were found during the COVID-19 pandemic and could be placed on a continuum: the Congruent-driven eater is at the functional pole of the continuum, whereas the Incongruent-driven eater is at the dysfunctional pole of the eaters continuum. In the middle of the continuum, the Incongruent-perceptual eater is at a critical crossing point. Significant differences were reported between eaters profiles.

**Discussion:**

The empirical results based on an eaters continuum conceptualization highlight the importance of understanding how people perceive their body to assess and promote food well-being.

## Introduction

From the outbreak of the SARS-CoV-2 virus leading to COVID-19 pandemic, physical and social distancing became part of daily life for millions of people around the world. Overall, scientific findings on COVID-19 are unequivocal: daily physical and social distancing can impact mental and physical health of populations (Cao et al., 2020; Ransing et al., 2021). More specifically, lockdown periods, social distancing, and reducing or even stopping non-essential activities created a real and deep transformation in daily living routines and participation in meaningful occupations (Cellini et al., 2020; King et al., 2020; Pekçetin and Günal, 2021). Between non-essential needs and essential needs, the complex act of eating became a source of distress, partly because fear of gaining weight was already pervasive and lockdowns led to increased availability of stock-up food at home, which could foster overeating and binge eating episodes (Phillipou et al., 2020; Scarmozzino and Visioli, 2020). People with eating disorders (EDs) have reported significant distress during this period of time (Flaudias et al., 2020; Rodgers et al., 2020; Touyz et al., 2020; Linardon et al., 2021). People with EDs reported negative impacts of the pandemic crisis on food concerns (e.g., food restriction, overeating/binge eating, purging, and over exercising) and body image concerns (e.g., perceived weight gain, fear of gaining weight, increase of body image disturbances; Fernández-Aranda et al., 2020; Termorshuizen et al., 2020; Schlegl et al., 2020a; Nisticò et al., 2021; Miskovic-Wheatley et al., 2022). A re-emergence of EDs symptoms has also been reported in former EDs patients (Castellini et al., 2020; Graell et al., 2020; Schlegl et al., 2020b; Miskovic-Wheatley et al., 2022). Research results are also raising concerns for the general population, reporting a marked increase of disordered eating behaviors such as meal skipping, overeating, binge eating, restrictive eating, and emotional eating among individuals with no history of EDs (e.g., Ammar et al., 2020; Cheikh Ismail et al., 2020; Di Renzo et al., 2020; Flaudias et al., 2020; Phillipou et al., 2020; Choukas-Bradley et al., 2022; Corno et al., 2022; Ramalho et al., 2022). An increasing number of studies published since the COVID-19 outbreak establish both an increase and a plurality of eating disorders in clinical population and disordered eating behaviors in the general population, especially in women and girls (Robertson et al., 2021; Schafer et al., 2022).

From an occupational perspective, many individuals’ eating behaviors were less regulated by social and organizational habits. Consequently, the daily routine could reflect the level of investment in certain occupations to the detriment or in favor of eating that is frequently observed in person with eating disorders (Christiansen and Townsend, 2010; Pierce, 2014; St-Pierre et al., 2022). In this article, we adopt the definition of occupation proposed by Schell and Gillen (2019). The term occupation refers to various daily activities, including personal care (looking after the self), leisure (enjoying life), and productivity (contributing socioeconomically; Schell and Gillen, 2019, pp. 124–140). The COVID-19 pandemic has changed the way different occupations are performed, which has involved disrupting roles and routines. Occupational balance is an important concept due to the direct relationship with health and wellness, and focuses on how occupations affect one another, whether occupations meet an individual’s needs, and time use in various occupations (Wagman et al., 2012). Occupational balance is an individual’s perceptual experience regarding the amount and variety of occupations (Wagman et al., 2012). In the context of the pandemic, individuals had to increasingly rely on perceptions related to habits associated with eating. Indeed, thoughts about food and body weight and shape could influence occupations (Tchanturia et al., 2013), as people reported having changed their main daily occupations associated with eating and occupations such as cooking. Concerning home cooking, a study reported that 49% of participants were more likely to consume fast food 1 to 2 times per week before the pandemic, while since the beginning of the pandemic up to 82% reported not consuming fast food (Husain and Ashkanani et al., 2020). Also, this study showed that there was an increase in the of participants who cooked for themselves, from 73 to 93%. Furthermore, the majority of participants perceived the benefit to consume healthy food by cooking for themselves (Husain and Ashkanani et al., 2020). Food perception and eating habits can be influenced by environmental cues and subjective beliefs (Oliveira et al., 2022). Studies have shown that the pandemic context has affected these dimensions and consequently food’s consumption and perception. For instance, food consumption could have been affected by unfounded beliefs about the possible transmission of the COVID-19 virus through food. Indeed, Faour-Klingbeil and colleagues’ study (Faour-Klingbeil et al., 2021) has shown that 70% of their sample was preoccupied that the COVID-19 could be a foodborne disease, together with concerns about the risk of being exposed to contaminated objects (e.g., food packaging), surfaces, and of coming across infected people during food shopping. In their experimental study, Oliveira et al. (2022) found that being exposed to a video with COVID-19 hygienic cues (i.e., a woman eating in presence of sanitary elements—such as hand sanitizer tube, medical face mask—and following hygienic measures, such as wear a face mask that is only removed after cleaning the hands with alcohol gel prior to food intake) led to decreased cravings and hedonic appreciation of the visual and olfactory food’s aspect, and lowered flavor expectations. Furthermore, after having watched the video with COVID-19 sanitary cues, participants’ appreciation of sweet food was significantly lower than after having watched percentage a non-pandemic video (Oliveira et al., 2022). Concerning physical activity, Bloom et al. (2020) found that 10% of their participants reported positive changes, while 20% reported negative changes. Wilke et al. (2020) examined preferences toward digital home exercise programs in 14 countries affected by COVID-19. The pandemic period was experienced as an opportunity to optimize one’s sport practice *via* homemade gym while escaping the gaze of others. Other studies highlighted a decrease of the quantity of physical exercise in adults (Galali, 2021), while in a study conducted by López-Moreno et al. (2020), 44.7% of participants did not perform any physical exercise during confinement. Beyond these self-reported physical activities, perceptions to weight gain or to fear of gaining weight led individuals to practice physical activity (Di Renzo et al., 2020).

Using videoconference technology for working and social activities increased significantly during the COVID-19 pandemic. Videoconferencing offers many advantages for maintaining social connection, workplace functioning, and well-being occupations, but individuals found themselves exposed to their own video reflection and opportunities to scrutinize their perceived physical appearance, which could contribute to the development of negative self-perceptions (Pino, 2022). Indeed, online platforms have affected the way people view themselves (Rice et al., 2021), so much that today some authors talk about the “Zoom dysmorphia disorder” (Pino, 2022). Furthermore, Vall-Roqué and colleagues’ study (Vall-Roqué et al., 2021) illustrated the association between social network use, body image disturbances (e.g., body dissatisfaction and body distortion), and low self-esteem among adolescents and young women following exposure to Instagram accounts focused on physical appearance. These results are consistent with Sajedi and colleagues’ study (Sajedi et al., 2021) on athlete students’ eating disorders focusing on dissatisfaction with different body parts and reflection on physical characteristics. Other than the emotional impact of the COVID-19 pandemic (in terms of anger or confusion; Pino, 2022), self-perceptions seem to have been considerably affected. A recent systematic review on the impact of COVID-19 on body image is unambiguous: COVID-19 pandemic had a significant impact on vulnerable people (Schneider et al., 2021).

Disturbances in the perceptions of body image and self-esteem were labeled by Cash and Smolak (2011) as distorted self-perceptions. These disturbances are associated with dysfunctional food intake, weight loss, and shape concerns; they are at the core of EDs (American Psychiatric Association, 2013). Distorted self-perceptions refer to the cognitive and affective dimensions of body image disturbances such as body shame and body dissatisfaction as well as other perceptual dimensions of body image disturbances (such as physical self-worth and physical self-appearance, Fox and Corbin, 1989). Together with body image-related disturbances and low self-esteem, Bruch (1962) highlighted long ago another dimension of disturbances in perception: a disturbance in the ability to perceive, be aware of, and correctly interpreting signals of the state of the body (e.g., signs of subjective and objective bodily pain and hunger and satiety), called perceptual disorders in EDs clinical population. These disturbances in interoceptive awareness and incongruence with engaging in adaptive eating behaviors have been identified as a central pathological mechanism in eating disorders, as well as for disordered eating behaviors (Fassino et al., 2004; Khalsa et al., 2015; Richard et al., 2019; Jacquemot and Park, 2020; DeVille et al., 2021). Healthy eating behaviors are associated with the congruence between noticing and appropriately responding to interoceptive signals of hunger and satiety, which is referred to as complete intuitive eating (Tribole and Resch, 1995; Tylka, 2006; Oswald et al., 2017). Intuitive eating is characterized by refusing both dietary restraint and the categorization of “bad” versus “good” food, and by unconditional permission to eat any food when hungry (Tribole and Resch, 1995). Intuitive eating appears to play the role of protective factor against disordered eating behaviors and it is also associated with more positive body image and emotional functioning, with greater weight stability, and lower weight (Avalos and Tylka, 2006; Tylka and Wood-Barcalow, 2015; Ruzanska and Warschburger, 2019; Christoph et al., 2021; Hazzard et al., 2021; Linardon et al., 2021; Messer et al., 2021). The increase in disordered eating behaviors during the COVID-19 pandemic could be associated with incongruent eating in response to emotional and external social signals, rather than a congruent response to interoceptive bodily signals and eating behaviors. For instance, boredom during lockdown periods could have promoted overeating as a dysfunctional strategy to escape monotony, and negative experiences related to the pandemic situation could give rise to restrained eating caused by a stress reaction that emulates the visceral cues related to satiety (Crockett et al., 2015; Corno et al., 2022; Sanlier et al., 2022). Pandemic-related stress could have also led to eating as a coping mechanism to face mood changes and negative emotions (Madali et al., 2021; Corno et al., 2022; Sanlier et al., 2022).

The COVID-19 pandemic imposed significant changes in daily routines and self-exposure. The scientific literature of the last 2 years has been highlighting detrimental effects of these changes on eating habits and self-perceptions in both clinical and general populations. However, to the authors’ knowledge, no study has explored and investigated the presence of eaters profiles during the COVID-19 pandemic. Identifying eaters profiles during the pandemic could help to promptly detect dysfunctional and unhealthy attitudes, cognitions, and behaviors and implement early interventions. Thus, the current study aims to explore and document eaters profiles in the general (Canadian) population in holistic perspective, including body perceptions and attitudes, eating behaviors (i.e., body image, behaviors, attitudes, and motivations regarding food) and occupations (i.e., physical activity and cooking) during the COVID-19 pandemic.

## Materials and methods

### Design and participants

Canada’s residents, French-speaking adults aged 18-year and older, were invited to participate to an online survey on behaviors, attitudes, and motivations regarding food and eating as well as body image and occupations during the COVID-19 pandemic. A total of 468 people provided free and informed consent to participate and allowed their data to be used anonymously. Only participants without missing data on the variables used to identify the clusters were kept for the analysis, for a final sample of 273 participants.

### Procedure

This cross-sectional study was conducted from 29th May to 1st September 2020. During this period, a health state emergency had been declared throughout Canada. During summer of 2020, Canada experienced a slight loosening of regulations. However, restriction on social life and leisure time (e.g., limitation to social gathering, physical distance, and mandatory use of masks), travels (e.g., restrictions for all non-discretionary travel), and work and school (e.g., home working) were still implemented to limit the spread of COVID-19. On August 2020, Canada entered the second wave of the pandemic and by the beginning of October 2020, most regions went into maximum alert (which involved closing non-essential businesses, restricting inter-regional travel, banning gatherings, curfew from 8 p.m. to 5 a.m., mandatory teleworking, and online school) (e.g., Gouvernement du Québec, 2021). The link to the online survey was shared through different platforms: websites, social network profiles, and community Listservs for students and professionals. This study has been approved by the ethical committee of the University of Quebec in Trois-Rivières (CER-20-266-10.21) and was carried out in compliance with current legislation regarding personal data protection (Helsinki Declaration of 1975, as revised in 2018, and the Tri-Council Policy Statement: Ethical Conduct for Research Involving Humans –TCPS 2 of 2018). 1). The objectives of the study were specified before data collection and the analytic plan was pre-specified.

### Assessment measures

*Sociodemographic questionnaire*. A set of items/questions aiming to measure sociodemographic profile of the sample was administrated to participants, including self-reported age, gender, height, and weight (to obtain Body Mass Index, BMI).

#### Body perceptions and eating attitudes and behaviors

Three questionnaires were used. First, the French-Canadian version of the Intuitive Eating Scale-2 (IES-2; Carbonneau et al., 2016; Tylka and Kroon Van Diest, 2013) was used to assess the degree of adherence to intuitive eating principles. This 23-item multidimensional self-report questionnaire comprises four subscales (i.e., Unconditional Permission to Eat, Reliance on Hunger and Satiety Cues, Eating for Physical Rather Than Emotional Reasons, and Body-Food Choice Congruence) and is based on a Likert scale from 1 “Strongly agree” to 5 “Strongly disagree.” In our sample, Cronbach’s alpha was 0.93, showing excellent internal consistency (Cicchetti, 1994). Second, the French very short version of the Eating Disorder Inventory (EDI-VSV 16 item) was used to assess disordered eating (Maïano et al., 2016). The questionnaire comprises eight subscales (i.e., Drive for Thinness, Bulimia, Body Dissatisfaction, Perfectionism, Interpersonal Distrust, Interoceptive Awareness, and Maturity Fears) and is based on a Likert scale from 0 “not at all” to 10 “extremely”. For the purpose of this study, only the body dissatisfaction and bulimia subscales were used. In our sample, Cronbach’s alpha was fair 0.72 (Cicchetti, 1994). Third, the Restraint subscale of the Eating Disorder Examination Questionnaire (EDE-Q; Fairburn and Beglin, 1994; Fairburn et al., 1993) was used to assess restraint attitudes and behaviors. The EDE-Q is a 28-item self-reported questionnaire adapted from the semi-structured interview Eating Disorder Examination (EDE; Cooper and Fairburn, 1987; Fairburn et al., 1993). The questionnaire comprises four subscales (i.e,, Restraint, Eating Concern, Shape concern, and Weight Concern), and its items are scored on a 7-point scale. Subscales and global scores of ≥4 indicate a clinical range (Carter et al., 2001; Mond et al., 2006; Carey et al., 2019). In our sample, Cronbach’s alpha was good 0.84 (Cicchetti, 1994).

#### Occupations

A range of descriptive information was recessed to assess occupations, as quantity of physical activity (including frequency and duration of activity in minute per week) and frequency of cooking (“1 = never cook,” “2 = cooking a few times a month,” “3 = cooking a few times a week,” and “4 = cooking everything that is eaten”).

### Statistical analyses

A cluster analysis was used to explore and determine eating profiles. A hierarchical clustering approach has been conducted using Ward’s linkage. Ward’s linkage uses an analysis of variance approach to evaluate the distance between clusters, the method attempts to minimize the Sum of Squares of any two possible clusters that can be formed at each step of the cluster analysis. Ward’s linkage is recognized as a very efficient hierarchical clustering approach. The cluster analysis was applied to the body perceptions and eating attitudes and behaviors scores. The number of clusters to retain has been determined with the Calinski-Harabasz pseudo-F index (Calinski and Harabasz, 1974). The Calinski-Harabasz pseudo-F index is a good complement to Ward’s linkage. Well-defined clusters have larger between-cluster variance and smaller within-cluster variance and Calinski-Harabasz pseudo-F index consider the ratio of between-cluster to within-cluster variance as well as the number of clusters and the number of observations. The optimal number of clusters maximizes the ratio of variance with respect to the number of clusters. The higher Calinski-Harabasz pseudo-F index indicates the optimal number of clusters. To look for differences between profiles for occupational characteristics, one-way ANOVA and Chi-square tests were conducted. Because there were unequal variances, Games-Howell *post-hoc* tests were calculated when occupational characteristics were significant. From Microsoft Excel spreadsheet, statistical analyses were performed using Stata 16.1 software for hierarchical clustering approach and IBM SPSS, version 27 for differences between profiles.

## Results

The sample was composed of 89.7% female (*n* = 245) and 9.2% male (*n* = 25) participants, whereas 1.06% (*n* = 3) participants self-identified as gender-fluid/two-spirit or do not want to state their gender or did not provide an answer to the gender question. Age ranged from 14 to 81 years old (M = 36; SD = 14.05). The mean BMI was 27 kg/m^2^ (SD = 8.9), ranging from 11 to 69 kg/m^2^.

### Descriptive interpretation of the clusters

The cluster analysis showed that three clusters are distinguished from each other according to the scores obtained on the measures of body perceptions and attitudes and eating behaviors. Table 1 shows information about each cluster. According to a comparison between the clusters within the sample of this study, Cluster#3 showed the lowest dysfunctional body perceptions and attitudes and eating behaviors according to the EDI and EDEQ and the highest intuitive eating scores according to IES. Cluster#1 showed the highest dysfunctional body perceptions and eating attitudes and behaviors and the lowest intuitive eating scores. Cluster#2 was characterized by scores halfway between the two other clusters, with a high level of body dissatisfaction score according to the EDI-BD. The eaters profiles were named based on the underlying constructs suggested by each cluster and the scientific literature (Fairburn and Beglin, 1994; Tylka and Kroon Van Diest, 2013): Incongruent-driven eaters (Cluster#C1), Incongruent-perceptual eaters (Cluster#C2), and Congruent-driven eaters (Cluster#C3).

**Table 1:** Body perceptions and attitudes and eating behaviors by cluster.

	Cluster#C1 Incongruent-driven eaters mean (SD)		Cluster#C2 Incongruent-perceptual eaters mean (SD)	Cluster#C3 Congruent-driven eater mean (SD)
EDI-BD	9.4 (1.41)		7.2 (2.08)	3.3 (1.23)
EDI-B	7.1 (1.94)		1.9 (1.72)	0.7 (0.86)
EDEQ-R	2.2 (1.65)		1.9 (1.79)	0.3 (0.28)
IES-UPE	3.0 (0.88)		3.1 (0.93)	4.0 (0.65)
IES-FCC	2.9 (1.05)		4.0 (0.75)	4.2 (0.63)
IES-EPR	2.1 (038)		3.2 (0.68)	3.7 (0.60)
IES-HSC	1.9 (0.71)		2.9 (0.90)	3.9 (0.81)

EDI-BD, body dissatisfaction subscale of eating disorder inventory; EDI-B, Bulimia subscale of eating disorder inventory; EDEQ-R, restriction subscale of eating disorder examination questionnaire; IES-UPE, unconditional permission to eat subscale of the intuitive eating scale-2; IES-FCC, body-food choice congruence subscale of the intuitive eating scale-2; IES-EPR, eating for physical reasons rather than emotional reasons of the intuitive eating scale-2; IES-HSC, reliance on hunger and satiety cues subscale of the intuitive eating scale-2; SD, standard deviation.

Table 2 shows sociodemographic characteristics and BMIs for the participants from each cluster. The interpretation of BMI using standard weight status categories shows that Congruent-driven eaters reported BMI between 16.9 and 57.6. kg/m^2^, with an average BMI of 24.9 kg/m^2^, corresponding to healthy weight (World Health Organization, 2020; Weir and Jan, 2021). The cluster of Incongruent-perceptual eaters reported BMI between 10.7 and 56.9 kg/m^2^, with an average BMI of 27.3 kg/m^2^, corresponding to overweight, and Incongruent-driven eaters reported BMI between 15.3 and 68.7 kg/m^2^, with an average BMI of 33.0 kg/m^2^, corresponding to class 1 obesity (World Health Organization, 2020; Weir and Jan, 2021).

**Table 2:** Clusters’ sociodemographic characteristics and BMI.

	Cluster#C1 Incongruent-driven eaters	Cluster#C2 Incongruent-perceptual eaters	Cluster#C3 Congruent-driven eaters
N (%)	38 (13.9%)	156 (57.1%)	79 (28.9%)
Female (%)	37 (97.4%)	146 (93.5%)	65 (82.1%)
Male (%)	1 (2.6%)	10 (6.5%)	14 (17.9%)
Other (%)	--	2 (1.3%)	1 (1.3%)
Mean age (SD)	38.5 (15.3)	35.9 (13.4)	35.8 (14.8)
Mean BMI (SD)	33.0 (11.3)	27.3 (8.0)	24.9 (8.5)

BMI, body mass index; SD, standard deviation.

### Occupations of the three eaters profiles

Tables 3, 4 show the occupational differences between each eaters profiles. Table 3 indicates the duration of physical activity for each cluster, in minutes. The analysis of variance shows a significant difference of quantity (frequency) of physical activity between eaters profiles [*F*(2) = 3.62, *p* = 0.028]. The Games-Howell *post-hoc* tests precise the significant differences, such as the cluster of Incongruent-driven eaters report significantly more quantity of physical activity than the cluster of Incongruent-perceptual eaters and the cluster of Congruent-driven eaters. However, no significant difference exists between Incongruent-driven eaters and Incongruent-perceptual eaters.

**Table 3:** Comparison of occupational-physical activity characteristics by clusters.

	Cluster 1 Incongruent-driven eaters mean (SD)	Cluster 2 Incongruent-perceptual eaters mean (SD)	Cluster 3 Congruent-driven eaters mean (SD)	F	*p*	Eta-Squared
Quantity of physical activity^*^	200.29 (126.68)	235.53 (281.00)	298.37 (413.90)	3.62	0.028	0.033
**Pairwise effects**	**Mean Diff.**	*p*	**SE**		**95% CI**	
					**LL**	**UL**
Cluster#C1 vs. Cluster#C3	147.51	0.005	46.03		37.89	257.13
Cluster#C1 vs. Cluster#C2	108.18	0.001	27.98		41.02	175.33
Cluster#C3 vs. Cluster#C2	−39.33	0.647	44.09		−144.31	65.65

* min per week; SD, Standard deviation; Mean Diff., Mean difference; *p*, Significance; SE, Standard error; LL, Lower limit; UL, Upper limit.

**Table 4:** Contingency table for occupational-cooking characteristic by clusters.

	Cluster 1 Incongruent-driven eater (%)	Cluster 2 Incongruent-perceptual eater (%)	Cluster 3 Congruent-driven eater (%)
Never cook	7.9	1.9	6.3
Cooking a few times a month	26.3	4.5	6.3
Cooking a few times a week	23.7	21.1	22.8
Cooking everything that is eaten	42.1	72.4	64.6

Table 4 indicates how frequently (in percentage) participants in each cluster cook. The results of the Chi-square analysis show that Incongruent-driven eaters profiles encompassed a statistically lower frequency of cooking than the other clusters [*χ*^2^(2) = 27.06. *p* < 0.001].

## Discussion

This paper aimed to explore and document eaters profiles during the pandemic in the general Canadian population in holistic perspective, including body perceptions and attitudes and eating behaviors (i.e., body image, behaviors, attitudes, and motivations regarding food), and occupations (i.e., physical activity and cooking). It is important to notice that these eaters profiles are not considered as a by-product from, of being caused by, the COVID-19 pandemic; instead, they inform on body perceptions and attitude, eating behaviors (i.e., body image, behaviors, attitudes, and motivations regarding food), and occupational habits that were observed during the pandemic.

### Eaters profiles in the context of the COVID-19 pandemic

This study highlights three distinctive profiles of eaters during the COVID-19 pandemic, and that could be placed on a continuum, from the pole of the Incongruent-driven eater profile to the opposite pole of Congruent-driven eater profile. The Incongruent-perceptual eater would be located in-between the poles.

*Congruent-driven eaters* have a positive relationship with their bodies. Those eaters reported on average higher scores in all the IES subscales, compared to the average scores reported by Tylka and Kroon Van Diest (2013) in their validation study. Thus, Congruent-driven eaters appear well-connected with their body signals of hunger and satiety. Their attitudes and eating behaviors are self-reported as driven by physical needs, they do not have forbidden foods, and they do not eat to regulate and reduce negative emotions. Congruent-driven eaters do not report engaging in disordered eating behaviors and attitudes such as bulimic and restrictive attitudes and behaviors. These results are in line with previous studies reporting intuitive eating to be associated with lower eating disorder symptomatology and lower body image concerns (Dockendorff et al., 2012; Tylka and Kroon Van Diest, 2013; Christoph et al., 2021; Hazzard et al., 2021; Linardon et al., 2021; Messer et al., 2021; Sanlier et al., 2022). Congruent-driven eaters practice more physical activity. Physical activity in Congruent-driven eaters could be driven by pleasure and not by appearance or feeling of guilt (Gast et al., 2015; Tylka and Homan, 2015).

*Incongruent-driven eaters*’ experiences appear disembodied; they seem less able to connect with their physical sensations of hunger and satiety. Those respondents reported lower average levels of intuitive eating than the ones reported by Tylka and Kroon Van Diest (2013). Incongruent-driven eaters seem not to be able to connect and trust their internal signals of hunger and satiety, and therefore they are more at risk of eating for emotional reasons and experience dietary restraint and weight gain (Birch and Fisher, 2000; Birch et al., 2003; Tylka and Kroon Van Diest, 2013). Indeed, the Incongruent-driven eaters self-reported to have the highest average BMI compared to the other two profiles. They reported not allowing themselves to eat unconditionally, a self-limitation well-known to be associated with experiencing guilt when eating, binge eating episodes, and overindulging in food (Polivy and Herman, 1999; Tylka and Kroon Van Diest, 2013). Indeed, the Incongruent-driven eaters reported high levels of bulimic attitudes and behaviors, and even if they do not reach the clinical cutoff at the EDEQ -R (i.e., a score of ≥4; Carey et al., 2019; Carter et al., 2001; Mond et al., 2006), they reported the higher levels of restrictive attitudes comparing to the Congruent-driven eaters and the Incongruent-perceptual eaters. Higher bulimic attitudes and behaviors could be explained by the dual-pathway model proposed by Stice (1994, 2001). Following this model, body dissatisfaction and bulimic behavior could be linked through two pathways: the dietary restraint and the negative affect pathway. The dietary restraint pathway proposes that body dissatisfaction results in restrained eating, which, in turn, could lead to overeating episodes. In the pathway of negative affect, body dissatisfaction could lead to negative emotions, which, in turn, could lead to overeating as a way to avoid feelings of aversion (Heatherton and Baumeister, 1991). Van Strien et al. (2005) extended the negative affect pathway in which the relation between negative affect and overeating could be explained by two intermediating variables: emotional eating and a lack of interoceptive awareness. Another possible explanation refers to the autoscopic overexposure of body. We could hypothesize that pandemic-related restrictions could have increased social media consumption and videoconferencing (Cooper et al., 2022), leading consequently to negative emotions about self-perception due to an autoscopic overexposure of body (Flaudias et al., 2020). Indeed, in Flaudias et al.’ (2020) study, a week after the perception of body overexposure, students developed “maladaptive eating behaviors” (i.e., binge eating and dietary restriction). Finally, Incongruent-driven eaters reported to practice physical activity and cooking less than the other profiles.

*Incongruent-perceptual eaters* seem to have some difficulties in connecting and listening to their bodily signals, but they are less disembodied than Incongruent-driven eaters. Their scores at the subscales of the IES are lower than the ones reported by Tylka and Kroon Van Diest (2013), with the exception of the Body-Food Choice Congruence subscale. Compared to the other two profiles identified in this study, Incongruent-perceptual eaters reported globally higher levels of intuitive eating and lower body dissatisfaction scores than Incongruent-driven, and higher scores of body dissatisfaction and lower scores of intuitive eating than Congruent-driven eaters. Body image disturbances, as body dissatisfaction, are recognized as a major risk factor for the development, maintenance, and relapse of eating disorders (Gardner, 2001; Dakanalis et al., 2017). Furthermore, the low global levels of intuitive eating warrant attention. Since they already show some issues in relation with their bodily experience, Incongruent-perceptual eaters could be at risk to slide toward the Incongruent-driven eaters profile. Thus, prevention programs should take into account these results in order to develop intervention targeting Incongruent-driven eaters.

### From eaters profiles to an eaters continuum?

**Figure 1:**
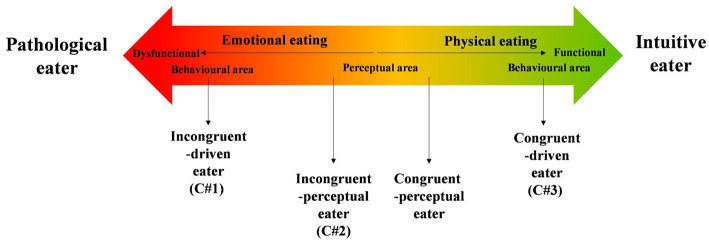
Proposed eaters continuum by perceptual-occupational-eating process.

Regarding the gradation of each feature that constitute the three eaters profiles, the results tend to support a dimensional approach of dysfunctional versus functional attitudes and eating behaviors (Pennesi and Wade, 2016; Monthuy-Blanc et al., 2021, 2022). Indeed, this approach echoes the actual quantitative and qualitative overall results of eating behaviors in COVID-19 literature (Ahuja and Banerjee, 2021; Robertson et al., 2021; Sanlier et al., 2022). These eaters profiles could be conceptualized on an “Eaters continuum” composed of two functional/dysfunctional processes resulting from the integration of perceptual and behavioral eaters (see “behavioral area” and “perceptual area” in Figure 1). More precisely, the functional pole could be understood as a combination of healthy and pleasant act of eating (Tribole and Resch, 1995), whereas the dysfunctional pole could be understood as a combination of unhealthy and unpleasant act of eating. The notion of combination is important because individuals with dysfunctional attitudes and eating behaviors can feel a state of pleasure after dysfunctional/unhealthy compensatory behaviors to relieve the negative emotions immediately (Cecchetto et al., 2021; Coulthard et al., 2021).

The intuitive eaters, on the functional end of the continuum, self-report cooking and eating with pleasure in manners that are congruent with their interoceptive cues. Pathological eaters, on the dysfunctional end of the continuum, self-report cooking and eating without pleasure or with unhealthy pleasure, depending on emotional needs and external signals that are incongruent with their interoceptive cues. In this study, the Incongruent-driven eaters profile falls in the dysfunctional behavioral area. Between these two poles, a “perceptual area” represents the large intermediate profile of transition from intuitive eaters to pathological eaters. In this area, the functional or dysfunctional nature of eating is not yet crystalized because the congruence or the incongruence between the body and food remains at the perceptual level and is not actualized at the behavioral level. For instance, the congruent-perceptual eaters (not found in our sample) would eat unhealthily but with self-compassion and based on perceptual signals. The Incongruent-perceptual eaters would present healthy eating habits together with emerging elements of guilt and uncompleted functional eating. The two poles of such eaters continuum conceptualization position functional eating as the ability to be perceptually connected, that is to perceive, be aware of, and correctly interpreting signals of the state of the body or not. In other words, functional eaters would have a better and healthier connection with their body mirroring perceptual congruence; dysfunctional eaters would have larger and unhealthier disconnection from the body (Perey and Cook-Cottone, 2020; Burychka et al., 2021).

### Strengths and limitations

This study gave a portrait on important dimensions of the daily life of the general population during the COVID-19 pandemic. One strength of this study is its holistic perspective, in terms of exploring perceptual, eating, and occupational dimensions. It could be interesting to further explore other aspects of the daily life of the general population during the COVID-19 pandemic, such as time and use of social network and actual use of videoconferencing, in order to explore the effect and relationship between the use of these technologies and perceptual, eaters profiles and occupational dimensions. This study shed light to two eaters profiles which present difficulties in connecting and understanding the internal signals coming from the body. Future studies should include specific measures targeting interoceptive awareness and interoceptive sensitivity (Richard et al., 2019; Jacquemot and Park, 2020; DeVille et al., 2021). It should be highlighted that the results of this study are limited by its cross-sectional design. That is, the results of this study give a portrait of eaters profiles during a specific time frame, and they do not inform about possible changes from pre-pandemic. Thus, this cross-sectional study does not allow us to interpret the results as reflecting causal relation between the COVID-19 pandemic (and its restrictions) and the emergence of these eaters profiles. Furthermore, since the sample was composed of a large majority of self-identified women, results may not be generalizable to people who self-identify with other genders. Future studies should include an equal number of individuals who self-identify with different genders in order to permit stratify subgroup analyses. Furthermore, studies should integrate both sex and gender assessment in order to explore and account for biological sex and gender differences and associations.

## Conclusion

This study on eaters profiles during COVID-19 pandemic reaffirms that beyond the biological ordinary act, eating is holistic and complex phenomenon closely associated with well-being and health. It is important to take account the plurality and evolution of eaters over time by conceptualizing an eaters continuum taking into consideration perceptual, eating, and occupational dimensions. To the authors’ knowledge, this is the first study that explores eaters profiles during the COVID-19 pandemic. Future studies should investigate eaters profiles and consider the possibility of other clusters and sub-clusters. Future studies on eaters profiles could guide the development and implementation of holistic prevention and treatment interventions, targeting perceptual, eating, and occupational dimensions.

## Data availability statement

The data for this study are available upon request addressed directly to the Research Ethics Boards (comite.ethique@uqtr.ca). The dataset is not publicly available due to privacy and ethical restrictions.

## Ethics statement

This study has been approved by the ethical committee of the University of Quebec in Trois-Rivières (CER-20-266-10.21) and was carried out in compliance with current legislation regarding personal data protection (Helsinki Declaration of 1975, as revised in 2018, and the Tri-Council Policy Statement: Ethical Conduct for Research Involving Humans –TCPS 2 of 2018). 1). The objectives of the study were specified before the data were collected and the analytic plan was pre-specified.

## Author contributions

JM-B and M-JS-P: conceptualization. JM-B, M-JS-P, and ÉT: methodology. JM-B and FB: data collection. JM-B, MR, GC, and SB: formal analysis. JM-B, GC, and SB: writing—original draft preparation. JM-B, GC, M-JS-P, SB, FB, LM-K, ÉT, and MR: writing—review and editing and supervision. JM-B: project administration and funding acquisition. All authors contributed to the article and approved the submitted version.

## Funding

This research was supported by the settlement fund of the Research Center of Mental Health University Institute of Montreal, Montreal, CAN, the RBC Royal Bank and Takeda Canada Foundations, 2021–2024 and MITACS-Acceleration awarded to the first author (JM-B); by a postdoctoral grant awarded to GC by the Fond de Recherche du Québec—Santé (FRQS; 289006); and by the Canada Research Chair in clinical cyberpsychology (950–210762) awarded to SB. The funders were not involved in the study design, collection, analysis, interpretation of data, the writing of this article or the decision to submit it for publication.

## Conflict of interest

SB is the President of, and owns equity in, Cliniques et Développement In Virtuo, a spin-off company from the university that distributes virtual environments designed for the treatment of mental disorders. The terms of these arrangements have been reviewed and approved by the Université du Québec en Outaouais in accordance with its conflict of interest policies. SB has received honoraria for presenting research and giving workshops. He also receives royalties from books.

The remaining authors declare that the research was conducted in the absence of any commercial or financial relationships that could be construed as a potential conflict of interest.

## Publisher’s note

All claims expressed in this article are solely those of the authors and do not necessarily represent those of their affiliated organizations, or those of the publisher, the editors and the reviewers. Any product that may be evaluated in this article, or claim that may be made by its manufacturer, is not guaranteed or endorsed by the publisher.
